# Mild Cognitive Impairment and Sarcopenia: Effects of Resistance Exercise Training on Neuroinflammation, Cognitive Performance, and Structural Brain Changes

**DOI:** 10.3390/ijms262211036

**Published:** 2025-11-14

**Authors:** Valeria Oporto-Colicoi, Alexis Sepúlveda-Lara, Gabriel Nasri Marzuca-Nassr, Paulina Sepúlveda-Figueroa

**Affiliations:** 1 Magíster en Terapia Física con Mención, Facultad de Medicina, Universidad de La Frontera, Temuco 4811230, Chile; v.oporto01@ufromail.cl; 2 Doctorado en Ciencias Mención Biología Celular y Molecular Aplicada, Facultad de Ciencias Agropecuarias, Universidad de La Frontera, Temuco 4811230, Chile; a.sepulveda23@ufromail.cl; 3 Departamento de Ciencias de la Rehabilitación, Facultad de Medicina, Universidad de La Frontera, Temuco 4811230, Chile; 4 Departamento de Ciencias Preclínicas, Facultad de Medicina, Universidad de La Frontera, Temuco 4811230, Chile

**Keywords:** resistance exercise, mild cognitive impairment, sarcopenia, neuroinflammation, hippocampus, neuroplasticity, dementia prevention

## Abstract

Mild cognitive impairment (MCI) and sarcopenia are prevalent age-related conditions that often coexist and share common mechanisms such as chronic inflammation, reduced neuroplasticity, and impaired muscle function. Resistance exercise training (RET) has emerged as a promising non-pharmacological strategy capable of addressing both physical and cognitive decline. The aim of this narrative review is to synthesize preclinical and clinical evidence on the effects of RET in older adults with MCI and sarcopenia, with a specific focus on its impact on neuroinflammation, cognitive performance and structural brain changes. At the molecular level, RET activates anabolic pathways, including PI3K/Akt/mTOR, enhances neurotrophic support via BDNF, NT-3, and IGF-1, and promotes hippocampal neurogenesis through exercise-induced myokines such as irisin and cathepsin B. RET also exerts immunomodulatory actions by shifting microglia toward anti-inflammatory M2 phenotypes, attenuating reactive astrogliosis, and supporting oligodendrocyte precursor cell differentiation, thereby improving myelin integrity. Neuroimaging studies consistently report preservation of hippocampal and precuneus gray matter, as well as improved white matter connectivity following RET. Clinically, RET has demonstrated significant and sustained improvements in executive function, memory, and global cognition, with effects persisting for up to 18 months. Collectively, RET represents a multifaceted intervention with the potential to delay progression from MCI to Alzheimer’s disease by integrating neuroprotective, anti-inflammatory, and anabolic effects. Standardization of RET protocols and identification of biomarkers of responsiveness are needed to optimize its role within multimodal dementia-prevention strategies.

## 1. Introduction

The demographic transition toward an aging population is accompanied by a sharp rise in age-related conditions, most notably dementia and physical frailty. Mild cognitive impairment (MCI), which affects approximately 10–20% of adults over 65 years, represents an intermediate stage between normal cognition and dementia [1,2,3]. Sarcopenia, with prevalence estimates ranging from 5% to 13% in community-dwelling older adults and up to 50% in institutionalized populations, reflects the progressive loss of skeletal muscle mass and strength [4,5]. Both conditions are highly prevalent among older adults and are significant determinants of disability, healthcare costs, and reduced quality of life [6,7,8]. Importantly, evidence suggests that MCI and sarcopenia frequently coexist—with reported co-occurrence between 8% and 20% depending on diagnostic criteria—highlighting the interconnected nature of brain and skeletal muscle aging [9,10].

The biological interplay between these conditions is increasingly recognized, with chronic inflammation, oxidative stress, and impaired trophic signaling emerging as shared mechanisms [11,12,13]. Physical exercise, particularly resistance exercise training (RET), has been proposed as a promising intervention that simultaneously targets muscular and cognitive decline [14,15]. While aerobic exercise has traditionally received greater attention, RET offers unique benefits related to muscle anabolism, myokine release, and modulation of neural plasticity [16,17,18].

Despite growing interest, the specific role of RET in mitigating neuroinflammation, preserving cognitive performance, and preventing structural brain changes in older adults with MCI remains insufficiently synthesized [19,20,21]. Therefore, this narrative review integrates current preclinical and clinical evidence on the effects of RET in older adults with MCI, with a particular focus on neuroinflammation, cognitive performance, and structural brain changes. By synthesizing mechanistic and clinical findings, we highlight RET as a promising multifaceted strategy to delay cognitive decline, counteract sarcopenia, and promote healthier trajectories of aging.

## 2. MCI

**Figure 1:**
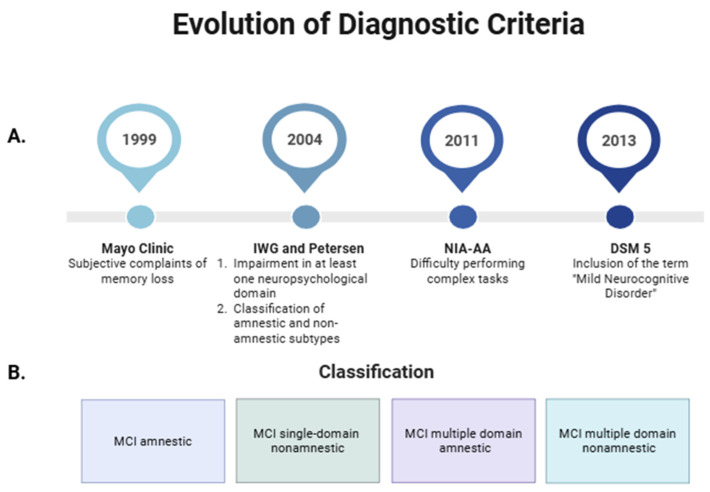
Overview of Mild Cognitive Impairment (MCI): Evolution of diagnostic criteria (**A**) and classification into amnestic and non-amnestic subtypes (**B**). Created in https://BioRender.com. IWG: International Working Group; DSM 5: Diagnostic and Statistical Manual of Mental Disorders, Fifth Edition; NIA-AA: National Institute on Aging and the Alzheimer’s Association.

The concept of MCI has evolved since its initial definition in 1999 by researchers at the Mayo Clinic, who characterized it as a subjective memory impairment without compromising activities of daily living (ADLs) [1,6]. Subsequently, in 2004, the International Working Group (IWG) on MCI broadened this definition, recognizing that cognitive impairment may involve additional domains such as language, complex attention, executive function, social cognition, and visuospatial abilities. Moreover, Petersen introduced the distinction between amnestic and non-amnestic subtypes [2]. In 2011, the National Institute on Aging and the Alzheimer’s Association (NIA–AA) proposed that mild alterations in ADLs may occur without a loss of functional independence [3]. Finally, the Diagnostic and Statistical Manual of Mental Disorders, Fifth Edition (DSM-5), redefined this condition under the term “Mild Neurocognitive Disorder”, distinguishing it from “Major Neurocognitive Disorder”, which corresponds to dementia (Figure 1A) [7].

The most widely used classification of MCI in clinical practice is based on the presence or absence of memory impairment, distinguishing between amnestic and non-amnestic MCI subtypes [2]. Additionally, depending on whether one or multiple cognitive domains are affected (as previously described), MCI can be subclassified as single-domain or multiple-domain. Accordingly, the subtypes include amnestic single-domain MCI, amnestic multiple-domain MCI, non-amnestic single-domain MCI, and non-amnestic multiple-domain MCI (Figure 1B) [22]. Currently, memory is defined as the ability to encode, store, and retrieve information [23], and it forms the basis for learning by recording information in the brain [24]. Since memory impairment is the core feature of the amnestic subtype of MCI, this condition is presumed to represent a prodromal stage of Alzheimer’s disease (AD). In contrast, the non-amnestic subtype seems to be more closely associated with progression to non-AD dementias, such as frontotemporal dementia, dementia with Lewy bodies, or vascular dementia [2,25,26]. Common signs and symptoms include forgetfulness, word-finding difficulties, difficulty performing complex tasks, and reduced ability to make decisions or plan activities. In addition, neuropsychiatric symptoms may also be present, such as depression, apathy, anxiety, irritability, sleep disturbances, agitation, and appetite or eating disorders [27]. These symptoms often coexist and are regarded as potential indicators of an increased risk of progression to dementia [25].

Epidemiological evidence indicates that within 4 to 6 years, more than 50% of older adults with MCI progress to dementia [25,26]. Importantly, progression rates can be even higher—up to 56%—in subtypes such as amnestic MCI, emphasizing that the risk of conversion largely depends on the specific type of MCI [26]. Therefore, this stage represents a critical therapeutic window for both prevention and intervention. According to a recent meta-analysis, the likelihood of cognitive stability was 49.8%, while 28.2% of individuals reverted to normal cognitive function [28]. Given that dementia is one of the leading causes of disability and dependency among older adults, early identification and appropriate management of MCI are essential for improving quality of life and reducing its impact on public health.

The high risk of progression from MCI to dementia, together with the heterogeneity of clinical trajectories, has prompted the search for biomarkers that allow for better prognostic stratification. In this context, the pathophysiology of AD is characterized by the abnormal accumulation of amyloid-β (Aβ) and neurofibrillary tangles (NFTs) in the brain, processes that begin decades before the onset of clinical symptoms [3,28]. On this basis, Braak staging and modern imaging techniques, such as Tau-PET, have emerged as valuable tools to predict, to some extent, the clinical course of individuals ranging from cognitively normal aging to dementia [29]. This approach relies on the pattern and extent of tau pathology described by Braak in six stages (I–VI), commonly grouped into three phases (I–II, III–IV, and V–VI), which provide relevant insights into the future risk of developing AD in patients with MCI [30,31]. These findings are obtained through the integration of neuroimaging techniques, mainly Tau-PET, which enables in vivo visualization of Tau accumulation, and structural magnetic resonance imaging (MRI), which reflects brain anatomical changes associated with disease progression [32]. As pathology advances across these stages, there is a progressive increase in the extent and density of Tau accumulation, which correlates with greater clinical severity and disease progression. Consequently, it is crucial to implement preventive strategies and timely diagnosis to reduce the impact of neurodegenerative diseases such as dementia. However, it has become increasingly evident that MCI progression cannot be fully understood without considering systemic factors beyond the brain itself. In this regard, sarcopenia has gained growing attention as a key age-related condition that may share pathophysiological pathways with cognitive decline, thereby contributing to the progression toward dementia.

## 3. Sarcopenia and MCI

Sarcopenia is a condition characterized by the progressive loss of skeletal muscle mass, strength, and function, which may result from an underlying disease (secondary sarcopenia) or be associated with age-related degeneration (primary sarcopenia) [29]. Regarding the mechanisms underlying primary sarcopenia, genetic and epigenetic alterations have been identified that impair cellular function during aging, affecting replication, transcription, and translation processes, and disrupting the balance between protein synthesis and degradation [30]. Epidemiological studies have reported a high co-occurrence of sarcopenia and MCI in older adults, with prevalence estimates ranging from 8% to 20% depending on diagnostic criteria [31,32]. Longitudinal data from community-based cohorts indicate that baseline sarcopenia is associated with a significantly increased risk of cognitive decline and incident dementia over 4–10 years of follow-up [4,5]. Conversely, the presence of MCI is linked to accelerated skeletal muscle mass and strength loss, and this effect is exacerbated in individuals with sarcopenic obesity, who exhibit increased systemic inflammation (chronic low-grade inflammation) and metabolic dysfunction, factors that accelerate both skeletal muscle loss and cognitive decline [8,33]. These findings underscore the urgent need for interventions that address both muscle health and cognitive performance in aging populations. This interaction not only exacerbates disability and dependency in older adults, but also highlights the importance of developing comprehensive, multidisciplinary strategies aimed at prevention, early detection, and targeted treatment to mitigate the burden of sarcopenia and cognitive decline.

### Crosstalk Between Skeletal Muscle and the Brain in MCI

In the context of MCI, growing evidence highlights the endocrine role of skeletal muscle, which secretes myokines that exert autocrine, paracrine, and endocrine effects, contributing to various exercise-induced adaptations [9]. These adaptations extend to skeletal muscle itself, adipose tissue, and the central nervous system (CNS), as myokine release supports communication between skeletal muscle and the brain [10,14,34]. Skeletal muscle regulates its own growth and repair through autocrine signaling mediated by growth factors and myokines. Factors such as insulin-like growth factor 1 (IGF-1) and fibroblast growth factor 2 (FGF-2) act locally on muscle fibers to stimulate protein synthesis, satellite cell activation, and tissue remodeling [35,36]. These autocrine mechanisms support muscle hypertrophy, regeneration, and metabolic adaptation in response to mechanical load and exercise. Beyond self-regulation, skeletal muscle communicates with neighboring tissues through paracrine signaling. Myokines like irisin, derived from fibronectin type III domain-containing protein 5 (FNDC5), enhance thermogenesis in brown adipose tissue, while interleukin-6 (IL-6) modulates local inflammation and lipid metabolism [15]. Together, growth factor-mediated autocrine signaling and paracrine myokine actions underscore the muscle’s central role in coordinating both its own homeostasis and systemic metabolic adaptations. Regarding myokines released in the brain, the most studied is brain-derived neurotrophic factor (BDNF), which has emerged as a key neurotrophin in this crosstalk [37]. Multiple exercise protocols have been associated with elevated peripheral circulating levels of BDNF in both animal models and humans [38,39,40,41], actively contributing to neuroplastic processes such as neuronal differentiation, growth, and survival [42,43]. BDNF plays a central role in enhancing spatial memory [44], cognitive performance [16], and learning [17], as well as promoting neurogenesis in key brain regions such as the amygdala, prefrontal cortex, and hippocampus [18,45,46]. Beyond BDNF and related neurotrophins, exercise-induced myokines have been implicated in muscle–brain communication. Among these, IGF-1 exerts fundamental neuroprotective functions [47,48,49]. It plays a role in preventing neuronal death, promotes hippocampal neurogenesis, and facilitates synaptogenesis [48]. Furthermore, IGF-1 supports the physiological phosphorylation of tau proteins and contributes to the clearance of Aβ peptides [49] processes intimately associated with the pathophysiology of neurodegenerative diseases. It has been shown that irisin, undergoes exercise-induced upregulation [50], crosses the blood–brain barrier, and stimulates BDNF expression in the hippocampus [51,52], which is linked to neuronal differentiation processes [53,54]. Similarly, cathepsin B, secreted during RET, promotes neuronal differentiation and migration [47,50,55], improving memory function by modulating Wnt and BDNF signaling pathways [56]. IL-6, when released transiently during exercise, exerts anti-inflammatory and neuroprotective effects by suppressing tumor necrosis factor-alpha (TNF-α) production and promoting the M2 microglial phenotype [57,58]. These additional mediators highlight the multifaceted nature of the endocrine role of skeletal muscle in maintaining brain health.

**Figure 2:**
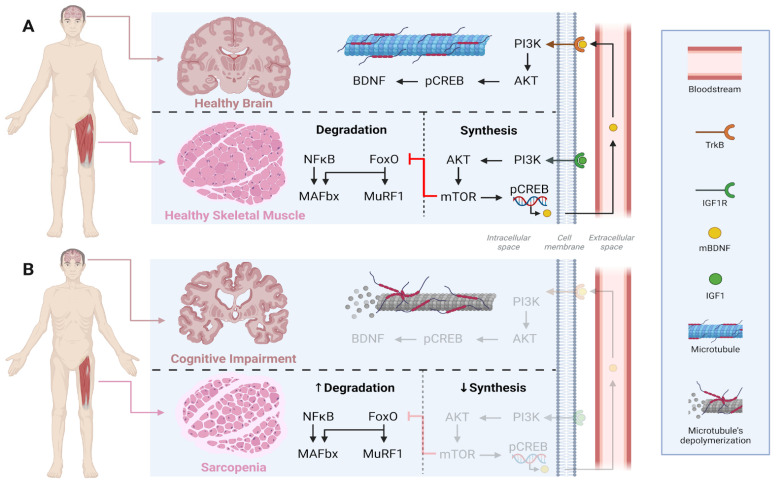
Comparison of crosstalk between skeletal muscle and brain in health (**A**) versus sarcopenia and cognitive impairment (**B**). Under healthy conditions, muscle contraction maintains a balance between anabolic (PI3K/Akt/mTOR, BDNF, IGF-1) and catabolic (NFκB/FoxO/MAFbx/MuRF1) signalings, supporting both muscle integrity and brain plasticity. In contrast, sarcopenia and cognitive impairment are characterized by reduced synthesis and increased degradation, leading to muscle atrophy and neuronal dysfunction. IGF1 = Insulin-like growth factor 1; IGF1R = Insulin-like growth factor receptor; PI3K = Phosphoinositide 3-kinase; Akt = RAC-alpha serine/threonine-protein kinase; mTOR = mammalian Target of Rapamycin; pCREB = Phosphorylated cAMP Response Element-Binding protein; mBDNF = mature Brain-derived Neurotrophic Factor; BDNF = Brain-derived Neurotrophic Factor; TrkB = Tropomyosin receptor kinase B; NFkB = Nuclear Factor kappa B; FoxO = Fokhead box O; MAFbx = Atrogin-1; MuRF1 = Muscle RING-finger protein 1.

Healthy skeletal muscle maintains protein homeostasis through the PI3K/Akt/mTOR signaling pathway activated by muscle contraction, which promotes phosphorylation of the transcription factor CREB (pCREB) and facilitates the release of mature BDNF (mBDNF) into the bloodstream [59]. Circulating mBDNF can cross the blood–brain barrier, bind to its high-affinity receptor TrkB, activate PI3K/Akt signaling in the brain, and promote endogenous BDNF expression [59]. Conversely, in older individuals with sarcopenia, skeletal muscle homeostasis is disrupted by reduced muscle contraction, leading to decreased stimulation of protein synthesis and increased proteolytic activity [60]. This imbalance is often linked to muscle disuse, reduced IGF-1 levels, impaired activation of the PI3K/Akt/mTOR pathway, and increased activity of the transcription factor FoxO, which drives catabolic processes. This process further induces the expression of muscle-specific E3 ubiquitin ligases, such as MuRF1 [61] and MAFbx/Atrogin-1 [62], which mediate protein degradation via the ubiquitin–proteasome system. As a result, sarcopenic muscle shows a diminished capacity to secrete myokines, including BDNF, impairing muscle–brain communication, accelerating cerebral atrophy, and worsening cognitive decline (Figure 2). Notably, the exercise modality par excellence for activating the PI3K/Akt/mTOR pathway is RET, which plays a pivotal role in preventing both sarcopenia [63] and MCI [64]. Moreover, RET has been shown to increase the gene expression of other neurotrophins such as NT-3 and NT-4/5 [65].

## 4. RET and Neuroinflammation

Neuroinflammation is a hallmark of nearly all CNS diseases and is increasingly recognized as a potential mediator of cognitive impairment [66]. Blood–brain barrier breakdown is also associated with faster cognitive decline, with inflammatory processes involving cell adhesion, neutrophil migration, lipid metabolism, and angiogenesis potentially contributing to this deterioration [20]. Neuroimaging studies increasingly support the role of neuroinflammation in the progression of dementia [67].

Microglia, the main innate immune cells of the CNS, play essential roles in surveillance, defense, and phagocytosis [21,68]. Their activity varies according to context, age, brain region, and metabolic demands [69]. Beyond their immune functions, microglia contribute to brain physiology by mediating synaptic pruning, regulating neuronal activity, and providing trophic support through factors such as neurotrophin-3, IGF-1, and nerve growth factor (NGF), which are critical for neuronal survival and development [68]. Importantly, microglia exhibit a wide range of phenotypes, transitioning between two major activation states: the classical pro-inflammatory M1 phenotype and the alternative anti-inflammatory M2 phenotype [70,71].

Astrocytes are the most abundant glial cells in the CNS and are crucial for maintaining neuronal homeostasis and brain function [72,73]. They regulate neurotransmitter clearance, ion balance, and provide metabolic support to neurons, mainly through glutamate uptake and lactate delivery as an energy substrate [73]. In addition, astrocytes contribute to synaptic modulation, neurovascular coupling, and blood–brain barrier integrity [69]. Depending on the context, astrocytes can acquire distinct phenotypes, ranging from the neurotoxic A1 subtype, induced by pro-inflammatory mediators, to the neuroprotective A2 subtype, associated with tissue repair and trophic factor release, including BDNF, NGF, and ciliary neurotrophic factor (CNTF) [72,73].

**Figure 3:**
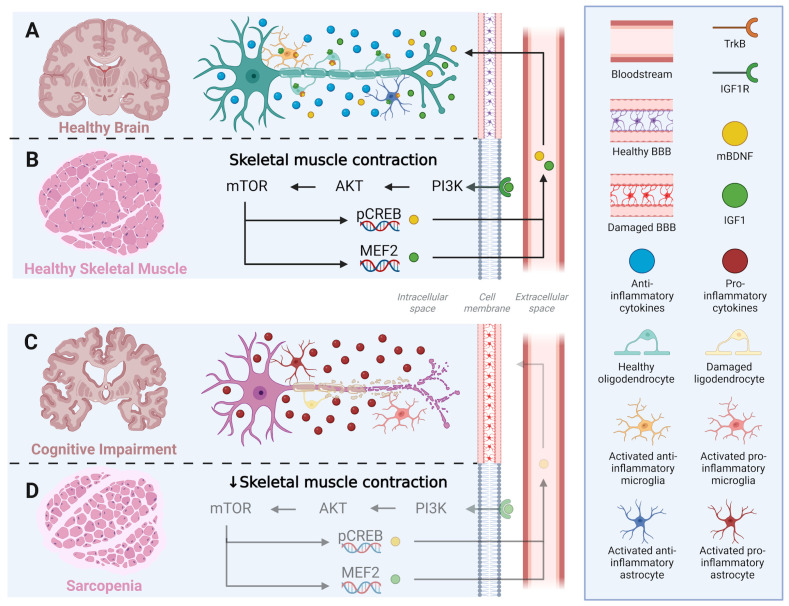
Crosstalk between skeletal muscle and brain, linking resistance exercise training to glial modulation and neuroinflammation. (**A**,**B**) Under healthy conditions, skeletal muscle contraction activates the PI3K–AKT–mTOR signaling cascade, enhancing the phosphorylation of CREB and activation of MEF2, which upregulate the expression of IGF-1 and mBDNF. These myokines cross the intact blood–brain barrier (BBB) and act on neuronal and glial cells through IGF1R and TrkB receptors, promoting anti-inflammatory microglial and astrocytic phenotypes (M2/A2) and maintaining oligodendrocyte integrity. (**C**,**D**) In contrast, sarcopenia and reduced muscle contractility impair PI3K–AKT–mTOR signaling, leading to lower myokine production, BBB dysfunction, and glial activation toward pro-inflammatory phenotypes (M1/A1). The resulting neuroinflammatory environment contributes to oligodendrocyte damage, demyelination, and cognitive decline. IGF1 = Insulin-like growth factor 1; IGF1R = Insulin-like growth factor receptor; PI3K = Phosphoinositide 3-kinase; Akt = RAC-alpha serine/threonine-protein kinase; mTOR = mammalian Target of Rapamycin; pCREB = Phosphorylated cAMP Response Element-Binding protein; mBDNF = mature Brain-derived Neurotrophic Factor; BDNF = Brain-derived Neurotrophic Factor; TrkB = Tropomyosin receptor kinase B; NFkB = Nuclear Factor kappa B; FoxO = Fokhead box O; MAFbx = Atrogin-1; MuRF1 = Muscle RING-finger protein 1.

Microglia and astrocytes do not act in isolation but interact dynamically to shape the neuroinflammatory milieu. Pro-inflammatory M1 microglia can induce the A1 astrocytic phenotype, amplifying synaptic loss and neuronal vulnerability, whereas M2 microglia promotes the protective A2 phenotype, fostering tissue repair and trophic factor release. In this regard, glial fibrillary acidic protein (GFAP) is widely used as the primary marker for astrocytes [72,73], while IBA1 is a standard marker for microglia [74]. This glial crosstalk is particularly relevant in MCI and AD, where chronic activation of M1 microglia and A1 astrocytes contributes to disease progression. Given this dual role of glial activation, interventions capable of promoting a shift toward the M2/A2 phenotypes, such as RET, may hold significant therapeutic potential in mitigating neuroinflammation-related cognitive decline (Figure 3).

### 4.1. Microglia and RET

M1 microglia secrete pro-inflammatory mediators, driving neuroinflammation and neurotoxicity, whereas M2 microglia release anti-inflammatory factors, contributing to neuroprotection [75]. Under normal conditions, microglia remain in a resting state. However, in response to pathological or neurotoxic stimuli or due to aging-related genetic changes, they can shift toward the classical M1 activation phenotype. This is triggered by exposure to lipopolysaccharides (microbe-associated molecular patterns, MAMPs), interferon-gamma (IFN-γ), and granulocyte–macrophage colony-stimulating factor (GM-CSF), leading to the release of pro-inflammatory molecules such as IL-1β, IL-6, IL-12, inducible nitric oxide synthase (iNOS), reactive oxygen species (ROS), TNF-α, major histocompatibility complex class II (MHC II), C-C motif chemokine ligand 2 (CCL2), C-X-C motif chemokine ligand 10 (CXCL10), and co-stimulatory molecules like CD86, CD16, and CD32 [21,68,69,76,77,78,79]. In contrast, alternative M2 activation is promoted by IL-4, IL-10, or immunoglobulin G (IgG), leading to the secretion of anti-inflammatory cytokines such as IL-10, transforming growth factor (TGF), CD206, suppressor of cytokine signaling 3 (SOCS3), found in inflammatory zone 1 (Fizz1), and YKL-40-like molecule 1 (Ym1) [21,68,69,76,77,78,79]. RET has been shown to downregulate IL-1β and TNF-α gene expression in muscle biopsies from healthy men after an acute session, while increasing IL-10 expression [80]. In healthy young and older women, a trend toward increased CD206 levels has been reported, reaching statistical significance in the older group [80,81]. Furthermore, aged mice subjected to RET exhibited reduced TNF-α, NF-κB, and IL-1β expression, along with increased IL-6 and IL-10 expression [82].

### 4.2. Astrocytes and RET

In the context of neuroinflammation, astrocytes undergo a process known as reactive astrogliosis, characterized by hypertrophy, proliferation, and upregulation of intermediate filament proteins such as GFAP [73,83]. A1 astrocytes, typically induced by pro-inflammatory cytokines such as IL-1α, TNF-α, and complement component C1q, secrete factors that can lead to synapse loss and neuronal death [84,85]. In contrast, A2 astrocytes, often activated by ischemic injury or anti-inflammatory signals, promote neuronal survival and tissue repair by releasing neurotrophic factors, including BDNF, NGF, and CNTF [84,86].

Importantly, astrocytic activation is not an isolated process but interacts dynamically with microglia. Activated microglia can induce the A1 phenotype via cytokine signaling, while astrocytes, in turn, can modulate microglial activation through the release of chemokines and anti-inflammatory mediators [85,87]. Exercise interventions, particularly RET, have been reported to attenuate reactive astrogliosis in aged rodent models, reducing GFAP expression and shifting astrocyte phenotypes toward a more neuroprotective profile [88,89]. Such modulation of astrocyte reactivity may contribute to the cognitive benefits observed with RET. Beyond these effects, RET also exerts a significant influence on oligodendrocytes and their precursor cells, enhancing remyelination processes and further supporting brain plasticity in the context of neurodegeneration.

### 4.3. Oligodendrocytes and RET

Beyond microglia and astrocytes, oligodendrocytes also play a crucial role in the neuroinflammatory environment of AD. Oligodendrocytes, the principal cells responsible for myelin formation in the CNS, perform functions that extend beyond myelination and axonal repair, as they also establish significant interactions with immune cells such as microglia [90,91]. During differentiation, these cells produce large amounts of membranes required for the formation of myelin sheaths, which are essential for wrapping and insulating neuronal axons, thereby optimizing the speed and efficiency of nerve conduction [92]. However, in the neuroinflammatory context characteristic of AD, the behavior of oligodendrocyte precursor cells (OPCs) becomes impaired, limiting their capacity for differentiation and myelin regeneration [76]. This effect is partly mediated by the disruption of growth factors and key signaling pathways, including PI3K and p38 MAPK [93]. RET activates key anabolic pathways such as PI3K/Akt/mTOR and MAPK/ERK, which stimulate the differentiation of OPCs into mature oligodendrocytes, thereby enhancing myelin synthesis and regeneration [94]. Recent evidence indicates that mechanical load during RET directly modulates oligodendrocyte lineage differentiation through mechanotransduction processes involving changes in cell structure and gene expression, where histone deacetylases (HDACs) convert mechanical signals into myelin-promoting responses [95,96]. Furthermore, mechanical stimulation promotes differentiation of OPCs through mechanosensitive signaling pathways involving focal adhesion kinase (FAK), integrins, and the transcriptional coactivators YAP/TAZ, which regulate cytoskeletal dynamics and nuclear mechanotransduction [96,97].

In addition, RET increases the release of neurotrophic factors, including BDNF and NT-3 with influence brain plasticity [98]. At the inflammatory level, RET inhibits NF-κB activation, decreases the production of pro-inflammatory cytokines (IL-1β and TNF-α), and enhances anti-inflammatory mediators (IL-10 and TGF-β), thus protecting myelin integrity [84,88]. Finally, RET improves mitochondrial biogenesis through peroxisome proliferator-activated receptor gamma coactivator 1-alpha (PGC-1α) and induces adaptive stress responses such as the upregulation of heat shock proteins (HSPs), contributing to proteostasis and strengthening oligodendrocyte resilience against neurodegeneration in AD [91,98].

## 5. RET and Brain Structural Changes

MCI is characterized by a general reduction in brain volume, particularly in the hippocampus and temporal regions, with losses estimated at 5–15% compared to healthy individuals [99,100]. The most pronounced differences are observed in the medial temporal lobe, particularly in the hippocampus, parahippocampal gyrus, and amygdala [99]. Studies utilizing MRI and advanced imaging techniques confirm a reduction in gray matter volume and structural abnormalities in white matter tracts within key brain regions. These changes correlate with cognitive decline, particularly in memory and executive function [100]. More specifically, studies have reported significant gray matter atrophy in the left amygdala and right hippocampus [101]. These regions are critically involved in emotion, cognition, and perception. In addition to these areas, MCI has also been associated with decreased gray matter in the thalamus and cingulate cortex [102]. The role of the hippocampus in MCI is particularly noteworthy, as hippocampal volume loss may serve as an early marker of neurodegenerative disease. In older adults with MCI, volume reductions in the CA1 subfield of the cornu ammonis and in subfields of the hippocampal subiculum have been identified as predictive markers of progression to AD [99]. Indeed, the gross volume of the left hippocampus, as measured by MRI, shows a negative correlation, indicating that smaller volumes are associated with greater cognitive decline in both MCI and AD [103].

Significant structural differences have been observed between the amnestic (aMCI) and non-amnestic (naMCI) subtypes of MCI. Specifically, aMCI is characterized by greater hippocampal volume loss—a region essential for memory—compared to naMCI [104]. Furthermore, patients with aMCI also exhibit greater cortical thinning, particularly in the entorhinal cortex and fusiform gyrus, highlighting the more pronounced nature of brain damage in this subtype [105]. In line with these observations, individuals with aMCI exhibit significant gray matter atrophy in two main brain regions when compared to cognitively healthy individuals: the left amygdala and the right hippocampus [104]. This is highly relevant, as the pattern of structural findings aligns with AD pathology, as defined by Braak staging, supporting previous research indicating that amnestic MCI progresses to AD at a higher rate than non-amnestic MCI. Finally, the progression of white matter hyperintensities (WMH) has been linked to an increased risk of cognitive decline in MCI, affecting functions associated with the frontal, temporal, and occipital lobes. Recent research has confirmed that individuals with MCI exhibit greater WMH volume than cognitively normal individuals [106]. However, it is important to note that no significant differences have been observed in the progression of WMH between individuals who eventually develop AD and those who do not. Similarly, no significant increase in the risk of conversion to AD has been directly attributed to WMH progression [104].

In transgenic rat models of AD, RET has demonstrated significant neuroprotective effects. Following a 4-week RET protocol, these animals showed improvements in object recognition, reduced escape latency, and fewer errors in the Y-maze test, indicating enhanced spatial memory and learning. Furthermore, using immunohistochemical techniques, a significant reduction in Aβ deposits was observed in the frontal cortex and hippocampus, thereby mitigating one of the key pathological hallmarks of AD [107]. Vints et al. [99] found no significant differences in hippocampal volume between the exercise and control groups in a 12-week RET program. However, notable findings included a 0.3% increase in the dentate gyrus volume in the exercise group, whereas the control group exhibited a 1.2% decrease, underscoring the potential neuroprotective effect of RET [99]. A recent study aimed primarily at investigating the impact of a 24-week RET program using a battery of cognitive tests and MRI yielded significant findings. A potential neuroprotective effect of RET was observed, as evidenced by preservation of gray matter volume in the hippocampus and the right precuneus. Additionally, the intervention group demonstrated greater white matter integrity in key structures, such as the corpus callosum, reflected by increased fractional anisotropy values compared to the non-intervention subjects [11].

### RET and Cognitive Performance

Beyond its well-established role in preserving skeletal muscle health, RET has emerged as a promising non-pharmacological strategy to counteract cognitive decline in aging populations. According to Zhang et al. [104] and Huang Xixiu et al. [93], RET is identified as the intervention with the greatest therapeutic potential for improving global cognitive function in individuals with MCI and dementia when compared to interventions such as aerobic exercise or multimodal training. Moreover, the positive effects are observed in training sessions conducted two or more times per week, with each session lasting more than 60 min [93,108]. Different authors [42,45,108,109] have demonstrated a statistically significant improvement in executive function resulting from RET, when compared to a control group (*p* < 0.05). Additionally, Singh et al. [19], in a study comparing various interventions over a 6-month period, found that RET significantly improved performance on executive function tasks, as assessed by the Wechsler Adult Intelligence Scale, Third Edition (WAIS-III) (*p* < 0.02). Furthermore, these effects were sustained at the 18-month follow-up [19]. Huang et al. [93], through a systematic review of randomized controlled trials, revealed that RET exerted a significant effect (*p* < 0.05) on memory function in individuals with MCI [93]. In accordance with these results, a recent clinical trial aimed at comparing the effects of a 24-week RET program on cognition reported significant improvements in the Rey Auditory Verbal Learning Test (RAVLT) following the intervention. These results reflect enhancements in episodic memory, verbal learning, and delayed recall capacity [11].

Other exercise modalities, such as high-intensity interval training (HIIT) or moderate-intensity continuous training (MICT), have shown effects on cognitive function; both have been reported to improve cognitive performance similarly to RET [12,110]. However, RET has a major advantage over other modalities due to its molecular effects, whereas HIIT or MICT primarily regulate energy metabolism and angiogenesis through SIRT/AMPK/PGC-1α signaling pathways [111], which are key signaling pathway for regulating energy expenditure [112]. In addition to enhancing cognitive function, RET also promotes protein synthesis, cellular growth [13,113], and healthier biological aging [114], which are key processes for preventing cognitive decline [115,116].

Recent literature has documented that the benefits observed after a single training session are primarily associated with improvements in specific executive functions, such as inhibitory control, working memory, and cognitive flexibility [117,118]. However, these findings remain limited and somewhat inconsistent, highlighting the need for further research to clarify the magnitude and underlying mechanisms of these acute effects [119]. 

## 6. Discussion and Conclusions

This narrative review integrates compelling evidence supporting RET as a dual therapeutic strategy to mitigate both sarcopenia and MCI through converging pathophysiological pathways. The mechanistic interplay between skeletal muscle and brain function, mediated by myokine signaling, neuroinflammatory modulation, and structural neuroplasticity, provides a robust biological rationale for RET’s efficacy in this population. RET has been shown to be effective in increasing skeletal muscle mass and strength regardless of age. At the molecular level, RET stimulates the PI3K/Akt/mTOR anabolic pathway, resulting in increased protein synthesis which in turn leads to skeletal muscle mass gains [59,63]. This activation, in turn, promotes the secretion of BDNF, providing neuroprotective mechanisms in the brain [33,37]. This dual action is particularly relevant given the established role of BDNF in hippocampal neurogenesis and synaptic plasticity [34,44]. The exercise-induced myokine irisin further amplifies these effects by crossing the blood–brain barrier and upregulating BDNF expression [51,52], while cathepsin B promotes neurogenesis by modulating Wnt signaling [56]. These findings position skeletal muscle as a critical endocrine regulator of brain health [9,10,34]. Therefore, RET acts both peripherally (improving appendicular muscles) and centrally (improving brain health).

The anti-inflammatory properties of RET represent another key mechanism underlying its cognitive benefits. RET promotes a shift from pro-inflammatory M1 toward anti-inflammatory M2 microglial phenotypes [115,116], reducing IL-1β and TNF-α while increasing IL-10 [47,48,49]. Concurrently, RET attenuates reactive astrogliosis, as evidenced by decreased GFAP expression [66,119], potentially preserving blood–brain barrier integrity and neuronal homeostasis [50,53]. These immunomodulatory effects are particularly relevant given the central role of neuroinflammation in MCI progression [11,12,111].

RET through imaging studies (MRI or CT-Scan) has demonstrated effects in preserving and/or increasing skeletal muscle mass, which favors preventing or combating sarcopenia. Currently, neuroimaging studies have also seen effects at the brain level. Preservation of hippocampal volume [11,99], particularly in the dentate gyrus [99], along with maintained gray matter density in the precuneus [11], aligns with observed enhancements in memory and executive function [19,93,108,109]. The increased fractional anisotropy in white matter tracts, including the corpus callosum [11], further suggests RET’s potential to maintain structural connectivity in aging brains.

From a clinical perspective, physical exercise has been shown to have benefits on cognition compared to no exercise. While several modalities of physical exercise (aerobic, concurrent, multicomponent, among others) have had positive effects on cognition, RET has demonstrated superior effects in improving global cognition in patients with MCI [65,108], particularly in executive function and memory domains [93,109]. Notably, these benefits appear sustainable, with effects persisting up to 18 months in follow-up assessments [19]. Importantly, as a person ages, they experience a decline in skeletal muscle mass and strength (sarcopenia) and cognitive decline (MCI), which can be accelerated by various factors. RET appears to be an effective strategy for both conditions. Some limitations warrant consideration. Variability in RET protocols (frequency, intensity, duration) across studies complicates direct comparisons [11,19,93,108,109]. The predominance of short-term interventions (≤6 months) in the existing literature [11,19] necessitates longer-duration trials to assess sustained effects. Additionally, most neuroimaging evidence comes from small samples [11,99], underscoring the need for validation in larger cohorts. Future research directions should focus on standardizing RET protocols for optimal cognitive outcomes, elucidating dose–response relationships, investigating combinatorial approaches (e.g., RET with cognitive training or nutritional interventions), and exploring biomarkers of RET responsiveness. But more importantly, it would be necessary to determine the minimum dose needed for an aging person to prevent the onset of sarcopenia and MCI.

In conclusion, RET emerges as a multifaceted intervention with the capacity to simultaneously target sarcopenia and MCI through activation of anabolic pathways, myokine-mediated neuroprotection, anti-inflammatory effects, cognitive performance, and structural brain preservation. The convergence of molecular, cellular, and clinical evidence underscores RET’s potential for integration into standard care protocols for older adults with sarcopenia and MCI. Ultimately, RET represents not only a preventive strategy, but also a promising therapeutic avenue with the potential to modify the trajectory of cognitive decline and delay progression to AD.

## Data Availability

No new data were created or analyzed in this study. Data sharing is not applicable to this article.
